# Genome-Wide Analysis of Alternative Splicing Identifies a Prognostic Signature in ER-Positive Breast Cancer

**DOI:** 10.3390/genes17091032

**Published:** 2026-08-28

**Authors:** Ahmed M. Basudan, Yazeed Alshuweishi, Hamood AlSudais, Mohammad A. Alfhili

**Affiliations:** Department of Clinical Laboratory Sciences, College of Applied Medical Sciences, King Saud University, Riyadh 12372, Saudi Arabia; yalshuweishi@ksu.edu.sa (Y.A.); halsudais@ksu.edu.sa (H.A.);

**Keywords:** alternative splicing, estrogen receptor-positive breast cancer, TCGA, survival, prognosis

## Abstract

Background/Objectives: Alternative splicing (AS) contributes substantially to transcriptomic diversity and has emerged as an important regulator of cancer progression. However, the genome-wide characterization of AS events specific to estrogen receptor (ER)-positive breast cancer remains limited. This study aims to comprehensively profile AS in ER-positive breast cancer and identify a prognostic AS signature associated with patient outcome. Methods: Clinical and splicing data (Percent Spliced In values) were obtained from The Cancer Genome Atlas (TCGA) for 737 ER-positive samples. Prognostic AS events were identified using Cox regression analysis. The Least Absolute Shrinkage Selection Operator (LASSO) model was used to construct an AS-based prognostic signature, and a standardized risk score was calculated for each sample. The signature was then evaluated by Kaplan–Meier (KM) analysis and receiver operating characteristic (ROC) curves, in addition to other methods to validate model performance. Furthermore, transcript-level annotation and RNA expression correlation were performed to evaluate biological relevance. Results: Profiling identified 6276 AS events across 4457 genes, with exon skipping (ES) representing the most prevalent class (34.4%). Model analysis established a novel five-event AS prognostic signature (comprising *DNAJC14*, *BAZ2B*, *PCDHAC1*, *PCDHA7*, and *DAPL1*). The signature significantly stratified patients into high-risk and low-risk groups for both disease-free survival (DFS; *p* < 0.001) and overall survival (OS; *p* < 0.001). HER2-specific analysis demonstrated more consistent performance in HER2-negative patients for both DFS (*p* < 0.001) and OS (*p* = 0.018). The model achieved area under the curve (AUC) of 0.804 for 60-month follow-up supporting long-term prognostic performance. Additionally, the signature demonstrated stable and reliable discrimination with a concordance index (C-index) of approximately 0.73 across multiple validation methods. Conclusions: The study identified AS signature with promising prognostic value in ER-positive breast cancer. This highlights the potential of splicing-based models to refine risk stratification beyond conventional gene expression analysis.

## 1. Introduction

Breast cancer is the most frequently diagnosed malignancy among women worldwide [1]. It represents a biologically and clinically heterogeneous disease with substantial variability in molecular characteristics, therapeutic response, and clinical outcome [2,3]. Among the molecular subtypes, estrogen receptor (ER)-positive breast cancer is the most common and accounts for approximately 70% of the cases [4]. Although ER-positive breast cancer cases generally exhibit more favorable outcomes than other subtypes, they demonstrate marked heterogeneity in recurrence risk, disease progression, and response to endocrine therapy [5,6,7]. Despite major advances in endocrine and targeted therapies, acquired resistance remain major clinical challenge with a substantial proportion of patients eventually developing relapse or metastasis [8,9,10]. This clinical heterogeneity highlights the need for additional molecular biomarkers that can improve risk stratification and identify patients at increased risk beyond conventional clinicopathological factors.

Large-scale genomic and transcriptomic studies have substantially advanced the understanding of breast cancer pathogenesis through the identification of somatic mutations, copy number variations (CNVs), gene expression signatures, and epigenetic alterations [11,12,13]. These approaches have provided important tools for disease classification, the prediction of prognosis, and therapeutic decisions [14,15,16,17]. Nonetheless, increasing evidence suggests that post-transcriptional regulation also plays a critical role in shaping tumor behavior and transcriptomic diversity. In particular, alternative splicing (AS) has emerged as a major regulatory mechanism that contributes additional transcriptomic and proteomic complexity beyond conventional gene expression analysis [18,19,20,21]. Unlike conventional gene expression analyses, AS captures transcript isoform diversity and therefore provides and an additional layer of biological information that may better reflect functional alterations driving tumor progression.

During AS, precursor messenger RNA (pre-mRNA) transcripts are differentially processed to generate multiple mRNA isoforms from a single gene [22]. This process includes acceptor splice site (ES), mutual exclusive exon usage (ME), alternative donor (AD) or acceptor splice site (AA), retained intron (RI), alternative promotor (AP), or alternative terminator (AT) [23]. Physiologically, AS contributes to normal cellular differentiation, tissue development, and the maintenance of cellular homeostasis [24]. However, dysregulated AS can alter cellular function and contribute to cancer development and progression. This can be through the generation of oncogenic isoforms and modulation of proliferation, metastasis, apoptosis, and therapy resistance [25,26,27,28].

Accumulating evidence indicates that AS plays an important role in breast cancer and may influence clinically relevant phenotypes [29]. In breast cancer, dysregulated AS has been associated with epithelial–mesenchymal transition (EMT), metabolic reprogramming, aggressive tumor behavior, and endocrine resistance [30,31,32]. However, most studies have focused on individual genes or mixed breast cancer cohorts, while genome-wide analyses specific to ER-positive disease remain limited. Because ER-positive tumors have distinct biological features, pan-cancer and mixed breast cancer cohort studies often overlook subtype-specific splicing alterations. Despite shared dependence on ER signaling and the availability of effective endocrine therapies, ER-positive tumors exhibit substantial heterogeneity in clinical response and long-term outcome, including the development of endocrine resistance and late recurrence. Subtype-specific analysis may therefore help identify splicing alterations that are associated with disease progression and clinical outcome within ER-positive disease. Moreover, the prognostic value of genome-wide AS events in ER-positive breast cancer for improving risk stratification remains incompletely understood.

The current study aims at the investigation of breast cancer-specific splicing changes in the cancer genome atlas (TCGA) to identify novel splicing events that define clinically distinct breast cancer ER-positive groups and improve risk stratification beyond the standard clinicopathological factors.

## 2. Materials and Methods

### 2.1. AS Data Acquisition and Processing

**Figure 1:**
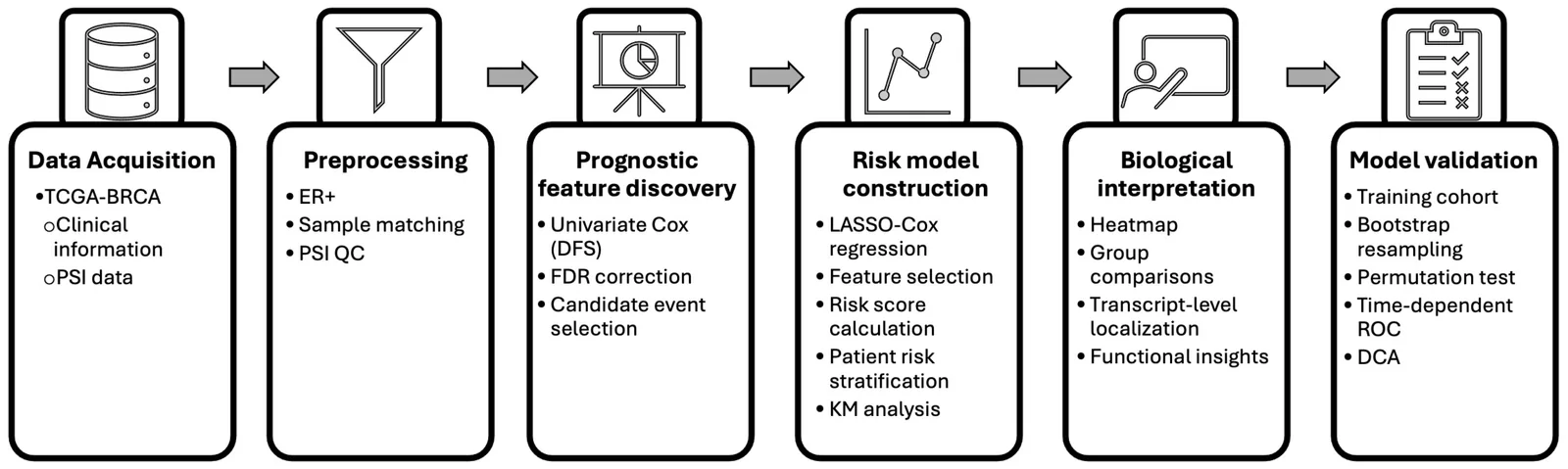
Overview of the study workflow. The figure shows the schematic illustration of the analytical pipeline used to identify prognostic alternative splicing (AS) events in estrogen receptor (ER)-positive breast cancer.

Clinical and survival data for TCGA-BRCA cohort were obtained from the “cBioPortal” [33]. Alternative splicing (AS) profiles were downloaded from “TCGASpliceSeq” [34] in the form of Percent Spliced In (PSI) values. These values represent the relative inclusion level of a splice event and range from zero to one. Seven AS event classes were included in the analysis: exon skipping (ES), mutual exclusive exon usage (ME), alternative donor (AD) or acceptor splice site (AA), retained intron (RI), alternative promotor (AP), or alternative terminator (AT). Clinical annotations were filtered to keep ER-positive samples only, based on ER immunohistochemistry (IHC) status. Samples lacking survival information were excluded. Clinical and PSI datasets were subsequently matched, resulting in a final cohort of 737 ER-positive tumor samples. An overview of data processing workflow is shown in Figure 1.

PSI matrices were imported and processed using custom scripts in R environment [35]. A unique identifier for each AS event was generated using gene symbol, AS class, and event ID. To improve analytical robustness and reduce unreliable events, AS events were subject to predefined quality control filtering criteria. Events were kept only if PSI values were available in at least 75% of tumor samples, with PSI standard deviation ≥ 0.10 and PSI range ≥ 0.1 across the samples. These thresholds were applied to remove low variability and events with limited biological relevance. After filtering, 6276 AS events across 4457 genes were used in downstream analyses.

### 2.2. Prognostic Feature Discovery

Cox univariate hazards regression analysis was performed for each AS event to identify prognostic events. PSI values were used as continuous variables and disease-free survival (DFS) was used as the primary clinical endpoint. Hazard ratio (HR) and 95% confidence interval (CI) with corresponding *p* value were calculated for each event. AS events with HR > 1 were considered risk-associated events, whereas events with HR < 1 were considered protective. To account for multiple testing, *p* values were adjusted using the Benjamin–Hochberg (BH) correction method.

### 2.3. Functional Enrichment Analysis

Gene Ontology (GO) enrichment analysis was performed using the “clusterProfiler” package in R [36]. Candidate genes were selected from the Cox analysis using the following criteria: *p* value < 0.01, β coefficient > 1, PSI standard deviation > 0.1. Events were classified as risk-associated when β > 0 and protective when β < 0. *p* values were adjusted using the Benjamin–Hochberg (BH) method with *p* < 0.05 as enrichment threshold.

### 2.4. Construction of Prognostic AS Signature

To construct a prognostic AS-based signature, top 300 AS events from the discovery analysis were selected according to *p* values and used for downstream analysis. Features with more than 10% missing PSI values were excluded, while the remaining missing values were imputed using median imputation. Least absolute shrinkage and selection operator (LASSO) regression was performed using the “glmnet” package in R [37]. The final model was selected using the more stringent λ_1se mode to reduce overfitting and improve model stability. A risk score for each sample was calculated as a weighted linear combination of PSI values and their corresponding coefficients. The risk score was subsequently standardized (z-score transformation), and patients were stratified into high- and low-risk groups using the median risk score as cutoff.

### 2.5. Survival Analysis

Kaplan–Meier survival analysis was used to evaluate the five-splicing event prognostic signature on the risk groups identified by the model using the “survival” package in R [38]. Differences in survival probability between groups were assessed using the log-rank test. Disease-free survival (DFS) was used as the primary endpoint for model evaluation, while overall survival (OS) was used for secondary survival analysis. To examine whether the model performance differed by HER2 status, additional analyses were performed for the HER2-positive and HER2-negative groups.

### 2.6. Transcript-Level Annotation

Transcript structural localization of the five prognostic splicing events was performed by integrating the TCGASpliceSeq annotations with additional transcript and exon annotations from Ensembl using the “biomaRt” package [39,40]. For each gene, a representative protein-coding transcript was selected based on coding sequence length and exon number. Transcripts maps were generated using “ggplot2” to visualize the location of the affected exon corresponding to each prognostic AS event [41]. Exon coordinates were transformed to display each transcript in the 5′ to 3′ orientation.

### 2.7. RNA Expression and PSI Correlation Analysis

RNA expression data for the genes included in the prognostic AS signature were obtained from UCSC Xena [42]. Expression values were represented as log2(RSEM + 1) normalized data. Comparisons between high- and low-risk groups were evaluated for each gene using Wilcoxon rank-sum test with Benjamin–Hochberg (BH) correction method. Correlation analysis between PSI and RNA expression were performed using the Spearman correlation test with reported rho and *p* values. Correlation patterns were visualized as scatter plots with fitted linear regression lines.

### 2.8. Model Validation and Performance Characterization

Multiple validation approaches were used to assess the robustness and predictive performance of the AS prognostic signature. Model discrimination using a train-split approach was implemented, where 70% of the cohort was randomly assigned to the training set and the remaining 30% to the test set. Internal validation was further evaluated using bootstrap resampling with 200 iterations. In addition, permutation analysis was conducted to estimate the likelihood of random performance. Concordance index (C-index) values were reported for all methods.

Time-dependent receiver operating characteristic (ROC) analysis was used to evaluate predictive accuracy at 12-, 36-, and 60-month time points, with the corresponding area-under-the-curve (AUC) values calculated. The potential clinical utility of the prognostic model was assessed using decision curve analysis (DCA). Predicted probabilities of progression estimated from the model were used to evaluate net clinical benefit across a range of threshold probabilities. Finally, model calibration was additionally evaluated by comparing predicted and observed DFS probabilities using calibration plots and correlation analysis.

### 2.9. Statistical Analysis

R environment was utilized for statistical computing and visualization (version 4.5.0) [35]. Heatmaps visualization was performed using “ComplexHeatmap” package [43], and PSI values were row-scaled to account for differences in PSI ranges across events. Group comparisons were visualized using box plot comparisons and analyzed with Wilcoxon rank-sum test. Forest plots to illustrate prognostic and magnitude of AS events, hazard ratios, and 95% confidence intervals were visualized using “ggplot2” [41]. Multiple-testing correction was performed using the Benjamin–Hochberg (BH) false discovery rate (FDR) method where applicable. Unless otherwise specified, statistical significance was defined as *p* < 0.05.

## 3. Results

### 3.1. Profiling AS Events in ER-Positive Breast Cancer

**Figure 2:**
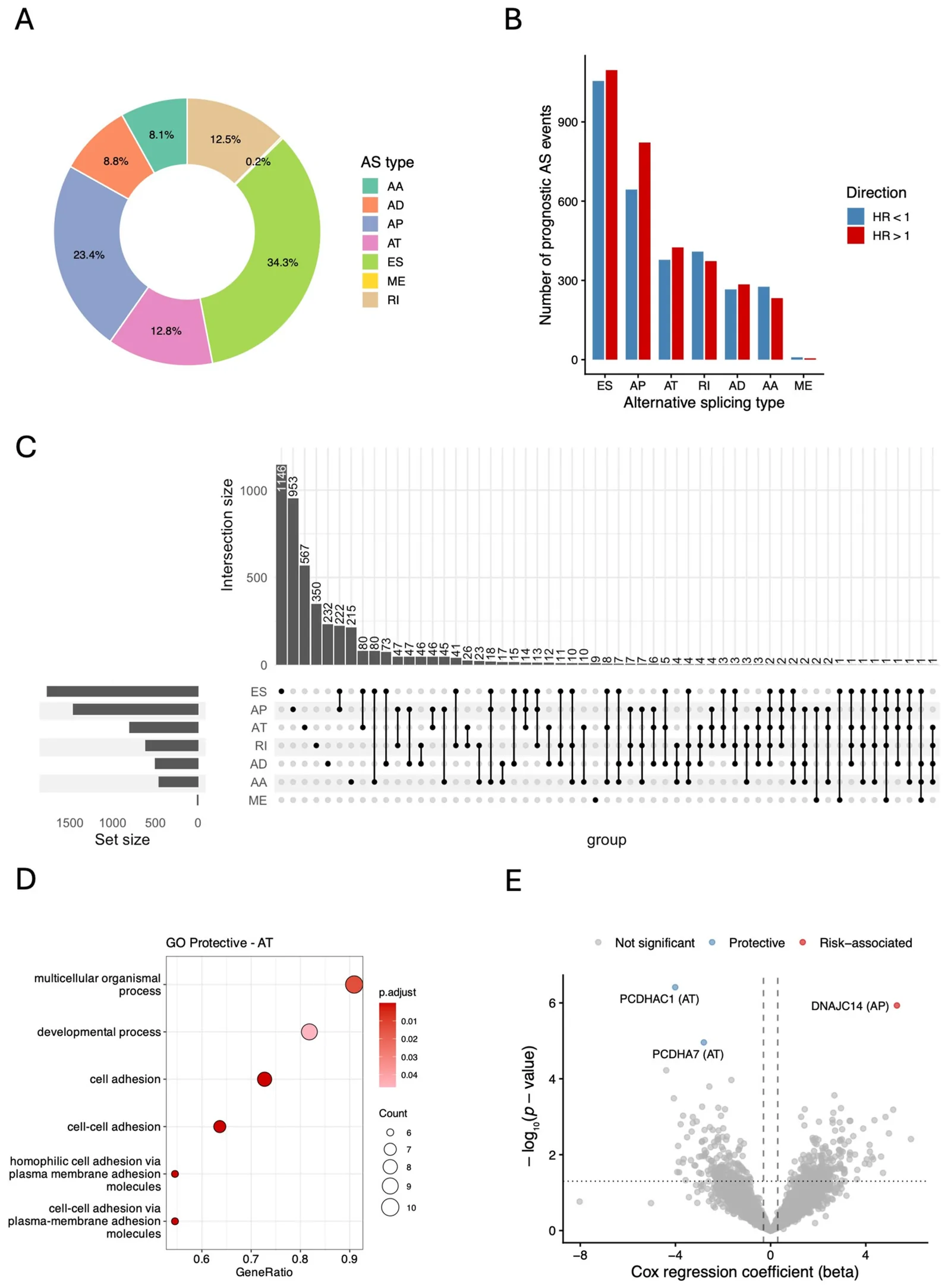
Profiling landscape of alternative splicing (AS) events in ER-positive breast cancer. (**A**) Distribution of seven classes of AS events detected after quality control filtering. ES, exon skipping; ME, mutually exclusive exons; AD, alternative donor; AA, alternative receptor; RI, retained intron; AP, alterative promoter; AT, alterative terminator. (**B**) Number of AS events for each class color-coded by hazard ration (HR) status. (**C**) UpSet plot showing the overlap of genes containing different AS event classes. Horizontal bars indicate the total number of genes associated with each AS class, whereas vertical bars represent the number of genes sharing combinations of AS event class. (**D**) Bubble plot of biological processes identified from the Gene Ontology (GO) enrichment analysis of AT-associated genes. (**E**) Volcano plot of significantly identified events from regression analysis. Blue dots represent protective features while red dots are risk-associated. Gray dots represent statistically insignificant events. *p* value and effect size cutoffs are indicated by horizontal and vertical dotted lines, respectively.

We characterized AS events among 737 ER-positive tumor samples. Clinical characteristics of those samples are summarized in Table 1. A total of 6276 AS events across 4457 genes passed the filtering criteria. The distribution of the AS events is shown in Figure 2A. Exon skipping (ES) was the predominant AS class, accounting for 34.3% of all detected events, consistent with previous reports [44,45,46].

To identify prognostic AS events, Cox hazards regression analysis was performed using PSI values as the continuous predictor and DFS as the endpoint. The numbers of AS events with favorable (HR < 1) or unfavorable (HR > 1) outcomes for each AS class are shown in Figure 2B and Supplementary Table S1. The distribution and intersection of the seven AS classes revealed that while the substantial proportion of genes exhibited a single dominant AS type, a notable fraction displayed multiple types of AS simultaneously (Figure 2C). As expected, ES was the most prevalent AS event.

Subclass-specific enrichment analysis of the AS events showed that protective AT-associated genes demonstrated significant enrichment for cell adhesion and development processes, particularly homophilic cell adhesion and cell–cell adhesion pathways (Figure 2D, FDR < 0.05).

Analysis of the regression model using stringent criteria (FDR < 0.05) identified three significant candidate events associated with prognosis (Figure 2E and Table 2). These included PCDHAC1|AT|73787 and PCDHA7|AT|73781 as protective markers, while DNAJC14|AP|22260 was a risk-associated marker.

### 3.2. Construction of Splicing-Based Prognostic Signature and Survival Analysis

**Figure 3:**
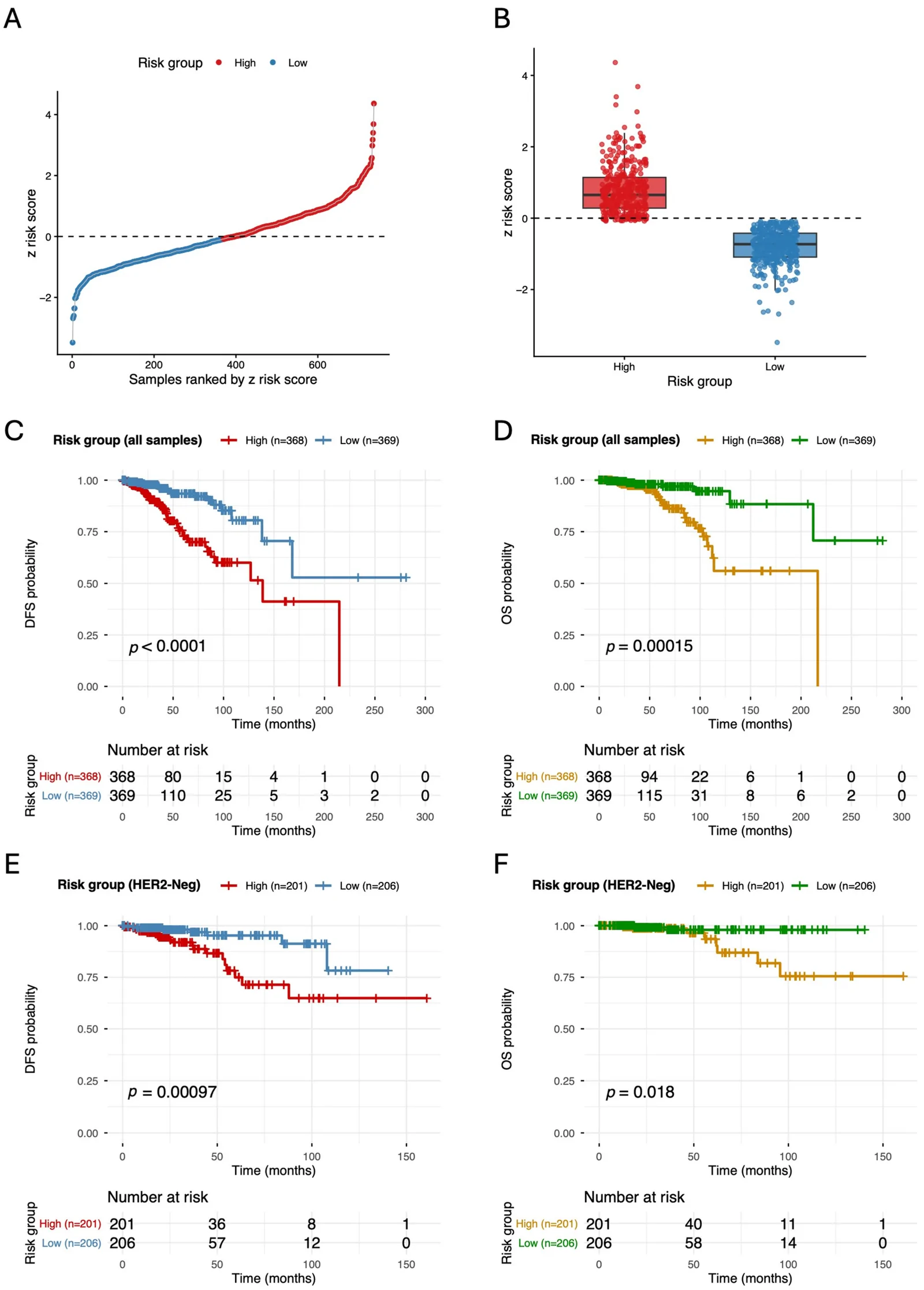
Survival analysis of prognostic signature identified from AS events. (**A**) Distribution of standardized risk scores. Samples are ranked by their calculated z score (red color denotes high-risk group while blue color represent low-risk group). (**B**) Box plot comparing the z scores of high (red)- and low (blue)-risk groups. (**C**,**D**) Kaplan–Meier (KM) plots of (**C**) disease-free survival (DFS) and (**D**) overall survival (OS) in ER-positive samples stratified into high- and low-risk groups according to the prognostic signature. Colors corresponding to risk groups are indicated. (**E**,**F**) KM plots of DFS (**E**) and OS (**F**) for the HER2-negative subgroup only. Log-rank *p* values are shown in each survival plot.

Top 300 AS events from the hazard analysis were retained for further LASSO analysis to construct a prognostic signature. A standardized risk score (z-score) was calculated, and samples were stratified into high- or low-risk groups (Figure 3A,B and Supplementary Table S2). The model identified five-splicing event prognostic signature for ER-positive tumors using DFS as the endpoint. The performance of both univariate and multivariate versions of the model was stable (Table 3).

The prognostic performance of the signature was then evaluated using KM survival analysis to assess DFS and OS among high- vs. low-risk groups. The splicing-based risk score was significantly associated with DFS (Figure 3C, log-rank *p* < 0.0001), indicating strong predictive performance. The KM analysis of OS demonstrated a similar trend, with high-risk patients showing significantly reduced survival probability compared to the low-risk group (Figure 3D, log-rank *p* = 0.0002). Given the clinical impact of HER2 status on disease prognosis [47], the analyses were further stratified accordingly to test HER2-negative samples only. The prognostic performance was stronger and more consistent in HER2-negative patients for both DFS (Figure 3E, log-rank *p* = 0.00097) and OS (Figure 3F, log-rank *p* = 0.018), whereas it was less stable in the HER2-positive group, likely due to limited sample size and events, in addition to distinct signaling pathways.

### 3.3. Biological and Transcript-Level Annotation of the Prognostic Signature

**Figure 4:**
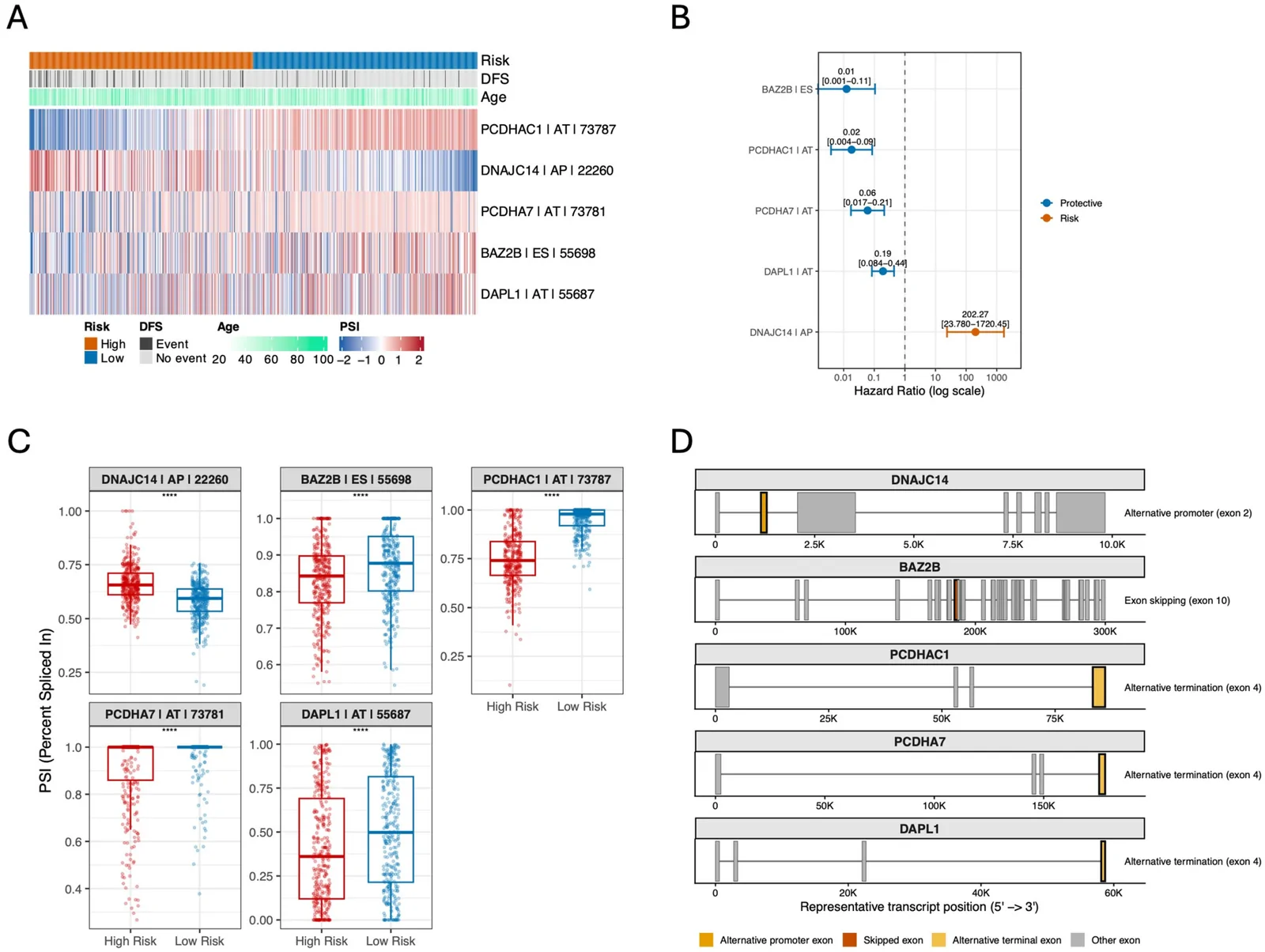
Biological characterization of the prognostic AS signature. (**A**) Heatmap showing Percent Spliced In (PSI) values of the prognostic signature events (rows) across ER-positive patient samples (columns) stratified by risk groups (high: orange; low: blue). The PSI color scale ranges from low (blue) to high (red). (**B**) Forest plot illustrating the hazard ratio (HR) on a log scale with corresponding 95% confidence interval of each AS event included in the prognostic signature. (**C**) Box plots comparing PSI values between high-risk (red) and low-risk (blue) groups for each prognostic AS event (**** denotes *p* < 0.0001). (**D**) Structural diagrams indicating the genomic configurations and the location of the affected exon or transcript region. The specific type of AS event is color-coded at the bottom.

To further characterize the biological relevance of the prognostic signature, transcript-level splicing patterns and their association with clinical risk were evaluated. As shown in the heatmap, distinct PSI expression patterns are seen across patients with clear differentiation between high- and low-risk groups (Figure 4A). The separation was most pronounced for the protective event PCDHAC1|AT|73787 and the risk-associated marker DNAJC14|AP|22260.

Prognostic direction and magnitude for each splicing event are illustrated in Figure 4B. Forest plot analysis revealed that DNAJC14|AP|22260 was strongly associated with poor prognosis and demonstrated the highest HR among all events (202.27; 95% CI: 23.78–1720.45). In contrast, the other four markers were associated with reduced risk and behaved as protective events (HR < 1).

Comparative PSI distribution analysis between high- vs. low-risk groups further supported these findings. As expected, the high-risk group exhibited significantly higher PSI values for DNAJC14|AP|22260 (Figure 4C; *p* < 0.0001). Conversely, the remaining four events showed significantly decreased PSI levels in high-risk patients relative to the low-risk group (Figure 4C; *p* < 0.0001). The consistent directional changes indicate that the prognostic model captures biologically coherent AS alterations associated with disease progression.

Transcript-level annotation revealed that the five prognostic events represented distinct classes of AS mechanisms (Figure 4D and Table 4). DNAJC14|AP|22260 involved alternative promoter event near exon 2, while BAZ2B|ES|55698 represented an exon skipping event affecting exon 10. The remaining three events, PCDHAC1|AT|73787, PCDHA7|AT| 7378, and DAPL1|AT|55687, corresponded to alternation events involving exon 4. Collectively, these findings suggest that the prognostic signature captures diverse AS mechanisms, including alterative promoter usage, exon skipping, and alternative termination, associated with ER-positive breast cancer progression.

### 3.4. Association Between Prognostic Splicing Events and RNA Expression

**Figure 5:**
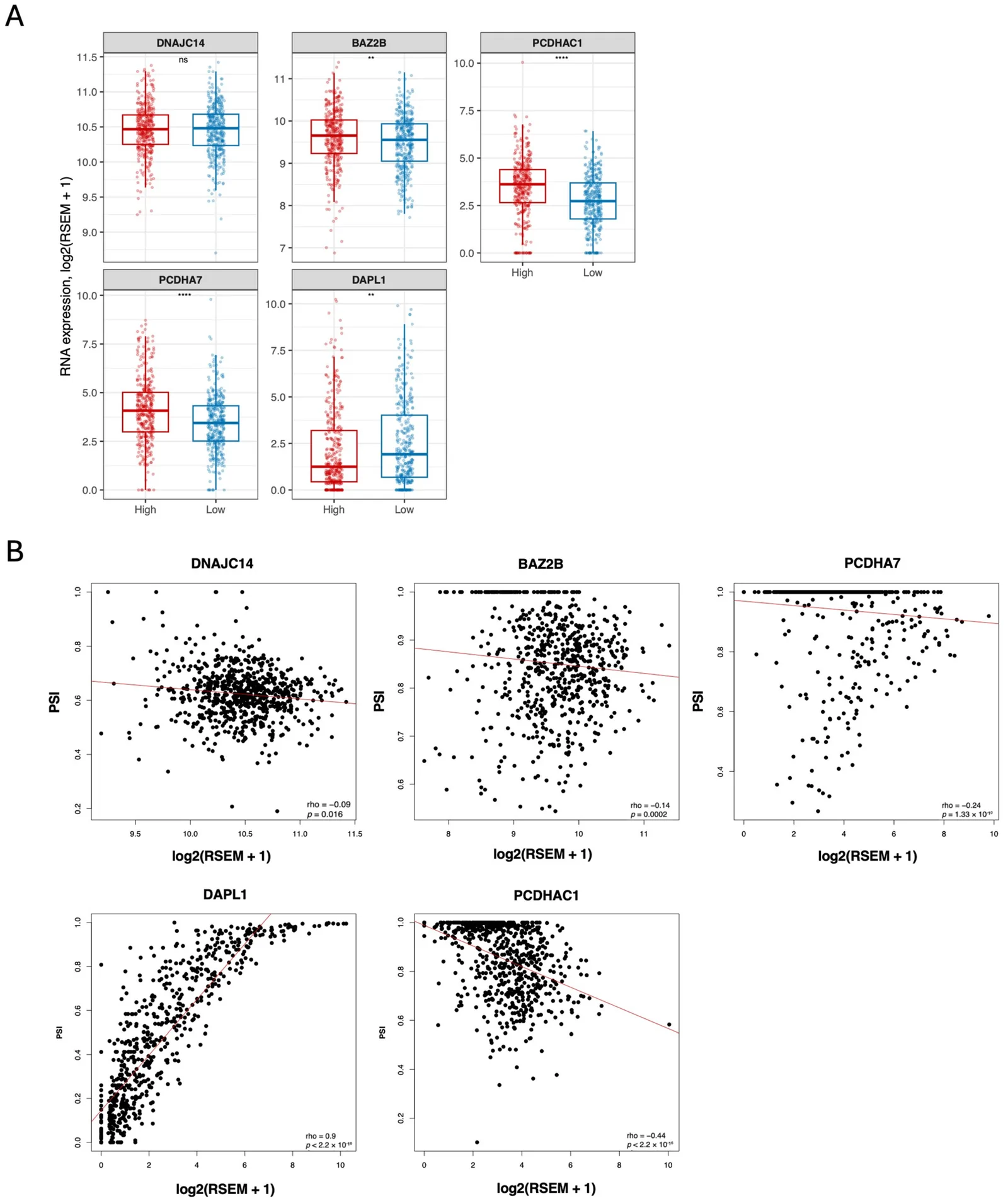
Association between prognostic AS evens and RNA expression. (**A**) Box plot comparisons of RNA expression between high-risk (red) and low-risk (blue) groups for the five genes included in the signature (ns denotes not significant, ** *p* < 0.01, **** *p* < 0.0001). (**B**) Correlation between PSI values and corresponding RNA expression for each prognostic gene. Red lines indicate the regression trend line. Correlation coefficients and associated *p* values are shown at the bottom of each plot.

To investigate whether the identified prognostic splicing events were associated with gene expression, the RNA expression levels of the five corresponding genes were analyzed across risk groups and correlated with PSI values. Expression analysis showed a significantly higher expression of PCDHAC1 and PCDHA7 in the high-risk group (Figure 5A; *p* < 0.0001). BAZ2B showed similar but modest significant increase in the same group (Figure 5A; *p* < 0.01), while DAPL1 was significantly increased in the low-risk group (Figure 5A; *p* < 0.01). DNAJC14 expression did not differ significantly between the risk groups.

Correlation analysis of PSI values and RNA expression revealed distinct relationships (Figure 5B). Significant inverse correlations were observed for PCDHAC1 (rho = −0.44, *p* < 0.0001), PCDHA7 (rho = −0.24, *p* < 0.0001), and BAZ2B (rho = −0.14, *p* < 0.001), with a weaker relationship for DNAJC14 (rho = −0.09, *p* < 0.05). In contrast, DAPL exhibited a strong positive correlation (rho = 0.90, *p* < 0.0001). These findings suggest that several splicing events may be associated with reduced transcript abundance, potentially reflecting isoform-specific regulatory effects.

### 3.5. Model Validation and Performance Characterization

To further evaluate the robustness and clinical unitality of the five-event AS prognostic signature, we assessed its discrimination, calibration, and decision-making performance. As illustrated in Table 5, the model demonstrated good discriminatory ability with a C-index of 0.73 in the training cohort, while bootstrap resampling (200 iterations) showed stable internal performance (C-index = 0.735 ± 0.04). Similar performance was observed in the test set (C-index = 0.733), supporting the generalizability of the model. In contrast, permutation testing produced a considerably lower C-index (0.536), confirming that the prognostic signal was unlikely to result from random chance.

**Figure 6:**
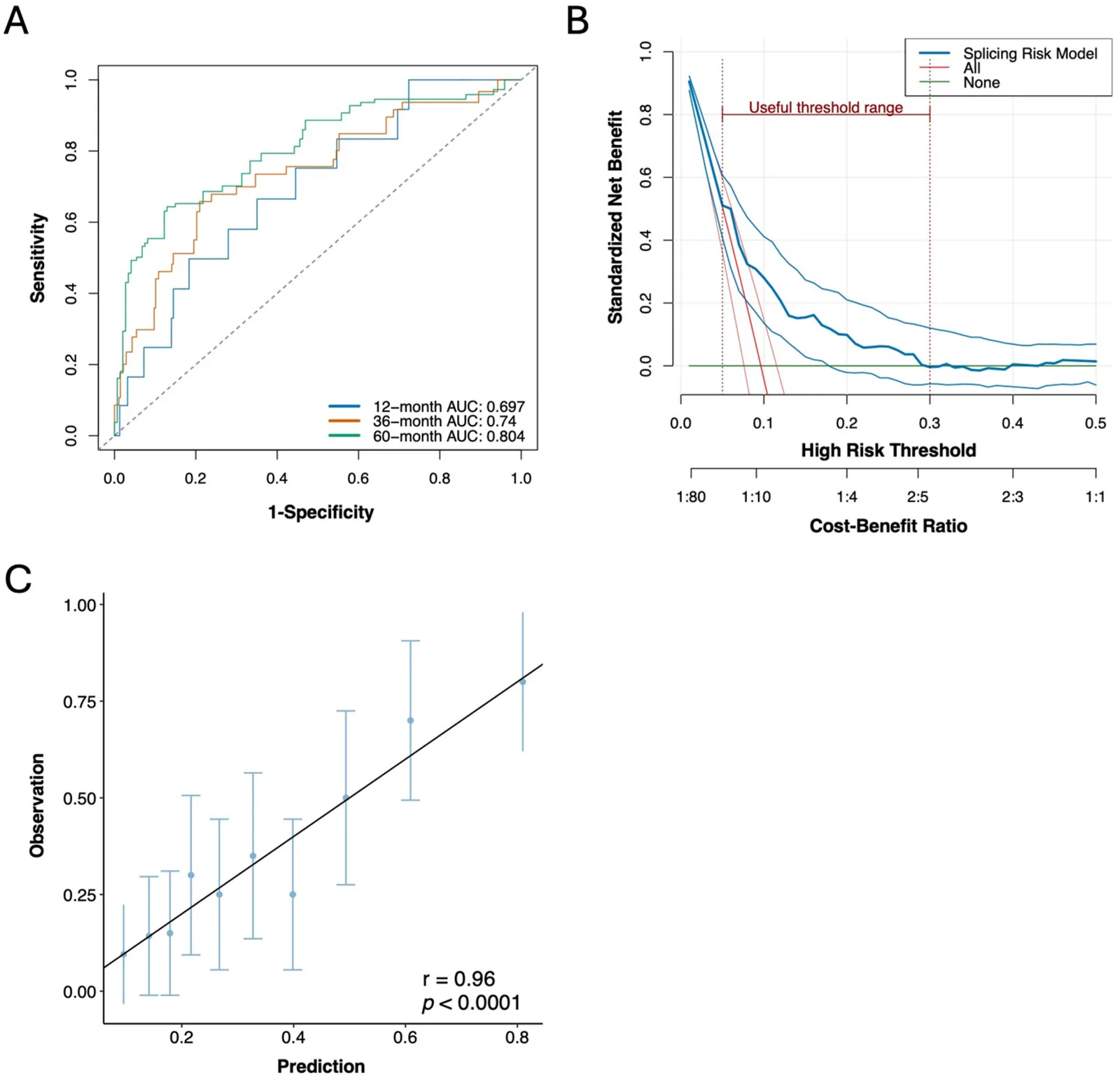
Performance evaluation of the prognostic risk model. (**A**) Receiver operating characteristic (ROC) curves evaluating the predictive performance of the model at three distinct follow-up intervals: 12 months (blue), 36 months (orange), and 60 months (green). Corresponding area under the curve (AUC) values are indicated. The gray dashed diagonal line represents the reference line for no discrimination. (**B**) Decision curve analysis (DCA) showing the net clinical benefit of the prognostic model (thick blue line) across a range of thresholds (thin blue lines represent 95% confidence intervals). The red and green lines represent the “treat all” and “treat none” strategies, respectively. A useful range is highlighted between the thresholds of approximately 0.05 and 0.30. (**C**) Calibration plot comparing predicted and observed DFS probabilities. Individual data points are plotted alongside their corresponding 95% confidence intervals (vertical bars). The diagonal black line indicates the linear association. Correlation coefficient and *p* value are shown at the bottom of the plot.

Time-dependent ROC analysis demonstrated that the model maintained consistent predictive performance across multiple follow-up intervals, with AUC values of 0.697, 0.740, and 0.804 for 12-, 36-, and 60-month DFS, respectively (Figure 6A). Notably, the predictive accuracy improved over longer follow-up periods, supporting long-term prognostic performance of the model.

Decision curve analysis (DCA) further demonstrated the potential clinical usefulness of the model (Figure 6B). The greatest clinical utility was observed within the approximate threshold probability range of 0.05–0.30, suggesting that the model may support meaningful risk stratification.

In addition, model calibration analysis showed good agreement between predicted and observed DFS probabilities around the reference line, indicating acceptable predictive uniformity (Figure 6C). The correlation between the two variables showed a strong positive association (r = 0.96, *p* < 0.0001), further supporting the reliability of the prognostic model.

## 4. Discussion

Alternative splicing (AS) is increasingly recognized as an important contributor to transcriptomic diversity and cancer heterogeneity [48,49]. While numerous studies have examined AS across mixed breast cancer cohorts [50,51,52] and pan-cancer datasets [29,53], subtype-specific investigations remain comparatively limited. In the current study, we performed a comprehensive analysis of AS events in ER-positive breast cancer and identified a five-event AS signature capable of stratifying patients into distinct prognostic groups. Our results indicate that AS alterations provide valuable molecular insights that are closely linked to disease progression and patient prognosis in ER-positive breast cancer.

A total of 6276 AS events across 4457 genes passed quality control filtering, highlighting the widespread occurrence of these alterations in ER-positive tumors. Among the seven AS classes evaluated, exon skipping (ES) was the most prevalent event type, accounting for approximately one third of all detected events. The finding is consistent with previous studies identifying ES as the dominant form of AS [18,54].

A key finding of this study was the development of the five-event AS prognostic signature capable of stratifying ER-positive breast cancer into clinically distinct risk groups. Patients classified as high-risk exhibited significantly shorter disease-free survival (DFS) and overall survival (OS) compared to low-risk patients. The association remained stable across multiple analytical approaches and was accompanied by multiple evaluation metrics. The prognostic performance of the signature appeared stronger in HER2-negative tumors than in HER2-positive disease. This may reflect a greater biological homogeneity of ER-positive/HER2-negative tumors, whereas HER2-driven signaling could influence the impact of splicing alterations. In addition, the smaller number of HER2-positive cases may also have limited statistical power.

Functional enrichment analysis revealed that protective alternative terminator (AT) events were significantly enriched in pathways related to cell–cell adhesion. Alternative termination may represent an effective means of regulating adhesion-related genes by generating transcript isoforms with distinct coding regions or 3′ untranslated regions, thereby influencing protein function, transcript stability, localization, or post-transcriptional regulation. Such transcript remodeling may help preserve adhesive cellular programs that limit tumor cell dissemination and are therefore associated with favorable prognosis. This finding is biologically relevant because the disruption of adhesion program is a fundamental feature of tumor progression and metastatic spread [55]. Cell adhesion molecules maintain tissue architecture and regulate intercellular communication, whereas the loss of adhesion facilitates cellular migration, invasion, and the acquisition of aggressive phenotypes. Consistently, two of the five signature components (PCDHAC1|AT|73787 and PCDHA7|AT|7378) belong to the protocadherin family of cell adhesion molecules. Protocadherins are known regulators of cell–cell interactions and tissue organization and have been implicated in tumor suppression, invasion control, and metastatic progression across multiple malignancies [56,57,58,59,60,61].

Consistent with previous studies demonstrating that AS events can correlate inversely with overall RNA expression [62,63], both protocadherin-associated events demonstrated protective prognostic behavior while showing inverse relationship between PSI values and transcript abundance. The remaining signature events further support the concept that transcript-level regulation contributes to breast cancer heterogeneity. DNAJC14|AP|22260 emerged as the strongest risk-associated event and remained significant after multiple-testing correction. Notably, overall *DNAJC14* RNA expression was not significantly different between risk groups, indicating that alternative promoter usage rather than total transcript abundance may drive its prognostic association. *DNAJC14* encodes a member of the Hsp40 (DnaJ) family of co-chaperone proteins. Although evidence linking DNAJC14 directly to cancer is limited, the dysregulation of the molecular chaperone network is increasingly recognized as an important contributor to tumor progression, cellular stress, and therapeutic resistance [64,65,66,67]. Another noteworthy component of the signature is the event BAZ2B|ES|55698. BAZ2B encodes a bromodomain-containing remodeling protein that regulates chromatin transcription. Given the close interplay between chromatin structure, RNA polymerase II activity, and co-transcriptional splicing, alterations involving BAZ2B may influence alterative splicing through the modulation of the chromatin landscape.

The clinical relevance of the identified signature was supported by multiple validation approaches. The model demonstrated consistent discriminatory performance across training, bootstrap, and test datasets, with C-index values around 0.73. Time-dependent ROC analysis further showed stable predictive performance across multiple follow-up intervals, with accuracy increasing at longer follow-up durations. Decision curve analysis suggested potential clinical utility with a relevant range of threshold probabilities. Calibration analysis demonstrated good agreement between predicted and observed outcomes. Overall, the findings indicate that the model possesses favorable prognostic characteristics.

Although the prognostic model demonstrated promising performance, some limitations still exist. The study relied on retrospective TCGA data and lacked external validation in an independent ER-positive breast cancer cohort. In addition, the biological roles of the identified events were inferred computationally and remain to be experimentally validated. Furthermore, the absence of comprehensive treatment and follow-up data restricted our ability to evaluate how therapeutic interventions may interact with risk stratification based on AS.

## 5. Conclusions

In conclusion, this study provides a comprehensive characterization of AS in ER-positive breast cancer and identifies a novel five-event signature associated with outcome. Our findings suggest that AS alterations may provide prognostic information complementary to conventional gene expression analysis and indicate a protentional involvement of adhesion-related splicing programs in disease progression. Further validation in independent cohorts is warranted in addition to experimental studies for functional validation and the clarification of the mechanistic roles of the identified events in ER-positive breast cancer.

## Figures and Tables

**Table 1:** Clinical characteristics of ER-positive breast cancer cohort.

Characteristic	Value (%)
Number of samples	737
Age	59 (49, 67) *
Menopause status	
Pre	177 (24%)
Peri	26 (3.5%)
Post	483 (66%)
Unknown	51 (6.9%)
Histology	
IDC	494 (67%)
ILC	178 (24%)
Other	65 (8.8%)
Stage	
I	128 (17%)
II	414 (56%)
III	173 (23%)
IV	8 (1.1%)
Unknown	14 (1.9%)
PR status	
Negative	113 (15%)
Positive	622 (84%)
Unknown	2 (0.3%)
HER2 status	
Negative	407 (55%)
Equivocal	137 (19%)
Positive	109 (15%)
Unknown	84 (11%)

Unless otherwise specified, data are shown as numbers with percentages in parentheses. Abbreviations: IDC = invasice ductal carcinoma; ILC = invasive lobular carcinoma; PR = progesterone receptor; HER2 = human epidermal growth factor receptor 2. * denotes median (Q1, Q3).

**Table 2:** Candidate events associated with prognosis.

Gene	Event ID	Splice Type	HR (95% CI)	*p* Value	FDR
*PCDHAC1*	73787	AT	0.02 (0.00–0.09)	<0.001	0.002
*DNAJC14*	22260	AP	202.27 (23.78–1720.45)	<0.001	0.004
*PCDHA7*	73781	AT	0.06 (0.02–0.21)	<0.001	0.023

**Table 3:** LASSO-Cox regression analysis of the splicing-based risk score.

Variable	HR (95% CI)	*p* Value
Univariate		
Risk score	2.50 (2.00–3.13)	<0.001
Multivariate		
Risk score	2.48 (1.98–3.11)	<0.001
Age	1.01 (0.99–1.03)	0.345

**Table 4:** Functional consequences of the five prognostic splicing events.

Gene	Splicing Type	HR Direction	Effect	Protein-Level Consequence
*DNAJC14*	AP	Risk (HR > 1)	Alternative 5′ start (different isoform)	N-terminal truncation or altered domain composition
*BAZ2B*	ES	Protective (HR < 1)	Internal exon removal	Altered protein structure upstream of bromodomain
*PCDHAC1*	AT	Protective (HR < 1)	Transcript termination (shorter isoform)	Loss of C-terminal region (likely cytoplasmic tail)
*PCDHA7*	AT	Protective (HR < 1)	Transcript termination (shorter isoform)	Truncated protein lacking intracellular domain
*DAPL1*	AT	Protective (HR < 1)	Transcript termination (shorter isoform)	Truncated protein or altered stability

**Table 5:** Validation and assessment of the splicing-based prognostic model.

Validation Method	C-Index	Purpose	Interpretation
Training cohort	0.73	Assess model discrimination	Good discrimination
Bootstrap (200 resamples)	0.735 ± 0.04	Internal stability validation	Stable performance
Test set	0.733	Assess generalizability	Consistent performance
Permutation	0.536	Negative control	Confirms non-random signal

## Data Availability

All data supporting the findings of this study are included in the article and its Supplementary Files. Clinical and survival data for TCGA-BRCA cohort were obtained from the “cBioPortal” (https://www.cbioportal.org/ “accessed on 10 December 2025”). Alternative splicing (AS) profiles were downloaded from “TCGASpliceSeq” (https://bioinformatics.mdanderson.org/TCGASpliceSeq/ “accessed on 16 February 2026”).

## Supplementary Materials

The following supporting information can be downloaded at https://www.mdpi.com/article/10.3390/genes17091032/s1; Table S1: AS events identified from Cox hazards regression analysis; Table S2: Calculated risk scores from LASSO analysis.

## Abbreviations

The following abbreviations are used in this manuscript:

AA	acceptor splice site
AD	alternative donor
AP	alternative promotor
AS	alternative splicing
AT	alternative terminator
AUC	area under the curve
BH	Benjamin–Hochberg
DCA	decision curve analysis
DFS	disease-free survival
ER	estrogen receptor
ES	acceptor splice site
FDR	false discovery rate
HR	hazard ratio
IR	intron retention
KM	Kaplan–Meier
LASSO	Least Absolute Shrinkage Selection Operator
ME	mutual exclusive exon usage
OS	overall survival
PSI	Percent Spliced In
RI	retained intron
ROC	receiver operating characteristic
TCGA	The Cancer Genome Atlas
